# Sepsis electronic surveillance and clinical outcomes: impact over mortality of a sepsis early detection electronic rule implemented in the emergency department

**DOI:** 10.1186/cc14024

**Published:** 2014-12-03

**Authors:** P Martin-Rico, A Valdivia-Perez, MD Marco-Lattur, J Chorda-Ribelles, N Lozano-Cortell, P Olcina-Lloret, R Andres-Navarro, E Mateo-Sanchis, M Jordan-Lluch, O Esparcia-Rodriguez, J Magraner-Egea, J Lacalle-Martinez, A Barcelo-Lopez

**Affiliations:** 1 Internal Medicine (Infectious D. Unit), Hospital de Denia, Denia Alicante, Spain; 2 Preventive Medicine, Hospital de Denia, Denia Alicante, Spain; 3 Microbiology Department, Hospital de Denia, Denia Alicante, Spain; 4 IT Department, Hospital de Denia, Denia Alicante, Spain; 5 Emergency Department, Hospital de Denia, Denia Alicante, Spain

## Introduction

Severe sepsis and septic shock (SS/SS) have a high mortality. Therapeutic guidelines can improve mortality, but early recognition and timely implementation of these requires a proactive attitude that can be electronically supported.

## Methods

From May 2013 our hospital implemented a Sepsis Code (SC) based on an early detection electronic rule developed by our multiprofessional sepsis team: clinicians and IT engineers (EMR Cerner Millennium platform) and a standardized order set plus systematic follow-up by our sepsis team. We performed a before-after study to assess the impact over mortality of this strategy. Time-series analysis of sepsis admissions and mortality from January 2011 to December 2013, before and after SC implementation. (Analysis by STATA.) All urgent admissions recorded in the minimum basic data set in patients over 14 years from 1 January 2011 to 31 December 2013 were included. Inclusion criteria: patients with ICD-9 sepsis-associated codes in the principal diagnosis or patients with infection-associated codes in the principal diagnosis together with sepsis-associated codes in secondary diagnosis. Medical records were manually reviewed by clinicians to confirm SS/SS diagnosis. Temporal series analysis (Poisson regression). First analysis: sepsis admissions in relation to total urgent admissions. Second analysis: deaths due to SS/SS related to admissions in this group. In both cases we compared results before SC activation (28 months) and after that (first 2 transitional months and 6 consolidated months). The multivariate adjustment in both analyses included year, month of the year, and months with activated rule. Graphic analysis estimated predictions for the last 8 months based on the previous 28 months, comparing both observed and predicted sepsis and deaths.

## Results

**Figure 1:**
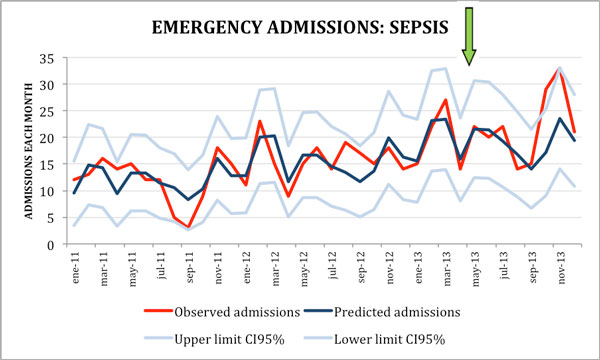
**Emergency admissions for sepsis**.

**Figure 2:**
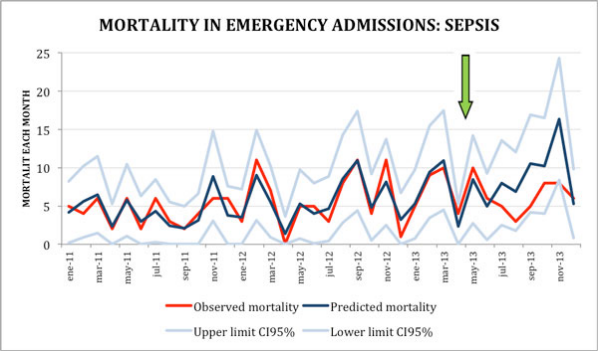
**Mortality in emergency admissions for sepsis**.

A total of 24,118 urgent admissions were included, 5,657 in the postalert period. Mean monthly admissions: 652 (SD 47) (570 to 740). In total, 408 and 178 SS/SS were identified in the prealert and postalert period, respectively. After SC implementation we observed no significant changes in sepsis admission risk but a clear downward trend in sepsis mortality: in the first 2 transitional months we did not observe major changes, while in the last 6 months the risk of death does fall 36% reaching statistical significance (IRR 0.64 (95% CI 0.43 to 0.97, *P *= 0.036)) (Table 1 Figures 1 and Figure 2). Both antibiotic door-to-needle time and adequacy significantly improved in sepsis cases where the alert was triggered.

**Table 1:** Risk of death in sepsis admissions

Variable	Category	IRR	95% CI	*P *value
Month	January	2.11	0.80 to 5.55	0.130
	February	2.55	1.04 to 6.24	0.040
	March	2.45	1.00 to 6.01	0.051
	April	1.0 (reference)		
	May	2.24	0.86 to 5.88	0.100
	June	1.45	0.53 to 4.01	0.470
	July	2.15	0.82 to 5.66	0.121
	August	2.62	1.00 to 6.85	0.050
	September	3.74	1.47 to 9.53	0.006
	October	2.31	0.89 to 6.02	0.085
	November	2.69	1.09 to 6.65	0.032
	December	1.89	0.71 to 5.01	0.203
Alert	Previous	1.0 (reference)		
	Transition	1.26	0.64 to 2.47	0.506
	Implemented	0.64	0.43 to 0.97	0.036

## Conclusion

Implementation of a SC triggered by an electronic detection alert, compared to the prealert period, decreased mortality risk by 36% (IRR 0.64 (95% CI 0.43 to 0.97, *P *= 0.036)) with the rule fully implemented.

